# Supplementary material for: Comparing immigration status and health patterns between Latinos and Asians: Evidence from the Survey of Income and Program Participation
Source: PLoS One. 2021 Feb 2;16(2):e0246239. doi: 10.1371/journal.pone.0246239 (PMC7853486; doi:10.1371/journal.pone.0246239)
Supplement: Attachment — Submitted filename: Response to reviews Submitted.docx [file pone.0246239.s001.docx]

**Reviewer 1**

**1. It would be helpful if the authors stated their expectation about the association between the level of US membership and health / insurance coverage in the introduction section. In this section, the authors wrote about the relative disadvantage of undocumented immigrants but rarely mentioned the reference group. For example, while they were clear that undocumented immigrants have lower median household income than the U.S.-born, they were not as clear when it comes to undocumented immigrants’ “lower access to health care”. Is the expectation that the association between US membership and health / insurance is approximately linear? If so, the authors should review not just the literature on undocumented immigrants but also on naturalized citizens/ LPRs / LNIs (they briefly mentioned this in the discussion section, but it would be helpful to elaborate it in the introduction as well). Or is the expectation that undocumented status presents a distinctive challenge to health and insurance coverage, and those who are documented (U.S.-born or LNI) are expected to be homogenous? If so, the authors should reconsider their categorization of US membership.**

We have added additional justification for considering the entire range of immigration statuses in our analyses. While previous studies compare undocumented immigrants to the US-born or other immigrant groups, we include a larger range of immigrant statuses to assess the argument from immigration scholars that incorporation outcomes, such as income, follow a gradient where the US-born, the most privileged and resourced group, have the best outcomes, followed by immigrant groups, such as citizens, legal permanent residents, immigrants with liminal statuses, and the undocumented. We expect our health outcomes to have similar pattern, with the US-born displaying the highest levels of health insurance coverage, followed by citizens, green card holders and the undocumented immigrants with the lowest levels. For poor self-rated health and disability, we expect US-born to have the lowest levels of these outcomes, reflecting their privileges and resources. Citizens, green card holders, and undocumented immigrants will have sequentially higher levels of these outcomes.

**2. The authors should explain how they chose the controls in their logistic regressions. The key results showed the association between race/ethnicity x immigration status and health/insurance net of not only age, gender and survey year, but also years in the U.S., education, marital status, region, and income. Why were these specific variables included? There certainly can be other observed and unobserved variables which are correlated with both race/ethnicity x immigration and health/insurance (e.g. occupation, rural/urban, health behavior…), and it is unclear whether they were excluded for a reason.**

We have added more justification to our covariates in the Methods section. We controlled for variables that would account for socioeconomic, immigration history, and demographic differences in both immigration status and our three health outcomes. For socioeconomic status, we chose education and income, and not occupation, because of missing data for older adults or those not in the labor force. For immigration differences we included duration in the United States. Duration in the United States varies by immigration statuses due to legal provisions (e.g. green card holders are not allowed to naturalize until after five years of US residence) and there are also well-known health differences by US duration. For demographic differences, we included age, gender, marital status, and US region of residence. We included region to account for geographical differences between traditional immigration gateways and new destinations. We did not include urban/rural, as there is probable correlation with geographic differences and because of the small number of Asian immigrants in rural areas. Finally, we did not include health behaviors because there few appropriate health behavior measurements in the SIPP. The SIPP is primarily an economic survey and there are no health behavior measures that would be appropriate to include as controls (i.e., exercise, smoking, etc.).

**3. Related to the issue above, the authors sometimes set up expectations in the introduction that did not seem connected with their analysis. For example, the authors wrote: “[w]e might expect differences by immigration status to be diminished among Asians compared to Latinos because their higher socioeconomic status (SES) and better health status overall”. It seems that the point of this sentence is to establish an expectation of how undocumented Asian immigrants fare in comparison to undocumented Latino immigrants. If that’s the case, then the authors should be showing regression results not controlling for SES.**

We have added two more models to show unadjusted differences between immigration statuses and our three outcomes. The first new model (Model 1) shows minimally adjusted differences in the main effects model and the second new model (Model 3) shows minimally adjusted differences in the interaction model. We have updated our results section to now include Models 1, 2, 3, and 4.

**4. Table 1 includes some descriptives that later did not appear as regression controls: household size, number of minors in household, and employment status. The authors did not describe these numbers in the text either. I suggest that they either highlight the importance of these descriptives or delete them.**

While we did not control for these covariates in the regression models, we keep them in Table 1 to provide a demographic picture of the Asian undocumented population, relative to other immigration status groups and to the Latino undocumented. This is the only paper that that we are aware of that has examined a large sample of Asian undocumented immigrants in depth and we want to provide baseline characteristics on this understudied sample.

**5. There is a typo on line 137: “pair”**

We have fixed this typo.

**6. What did the authors do about missingness on covariates? Did they also use multiple imputation or did they use a smaller sample? These could be clarified in the methods section.**

We handled all missing data the same way. We used Stata’s mi command, which uses multiple chained equations (MICE) to estimate respondent-level missing data on all variables. This approach assumes that variables are missing at random (MAR) and that other variables in the dataset can be used to predict the missing variable. We used MICE to first handle all missing data besides migration information. We averaged across ten imputed datasets to create a single dataset that was only missing migration information. We conducted MICE again for the migration information only and created ten datasets with imputed migration information that were combined in our analyses using Rubin’s rules.

Reviewer 2
**1. It sounds like the method for determining undocumented status may miss visa overstayers, if people who enter as non-immigrants and have not adjusted their status are coded as LNIs. Is it possible to look at the years in certain statuses, especially tourist visa, to see if they have overstayed the time typically granted on the I94 (six months max, I think)?**

The reviewer is correct that people who may have overstayed their visas will be counted as LNIs. We have re-analyzed our results with LNIs who have over 6 years residence in the United States recoded as undocumented. We chose 6 years as the cut-off for LNIs because of the policies that require LNIs (e.g. refugees) to adjust their visa status by this time. After making this adjustment, LNIs constituted less than 2% of either the Latinos or Asians sample and we decided to drop them from the analyses.

**2. Is 2% undocumented among Asians what would be estimated given the 1.5 million undocumented Asians estimate by MPI? Compare to validate the procedure for Asians. I was also surprised by the higher rate of marriage among undoc Asians, in spite of their younger age. Thoughts on this? Can you refer the reader to other studies that use the SIPP to look at undoc Asians?**

After making our coding change, the proportion of undocumented Asian immigrants in the SIPP rose from 2.6% to 8%. This is closer, but still lower than other estimates. The Center for Migration Studies, for example, estimates that 15% Asian immigrants are undocumented. We suspect the diverging estimates are from the different sampling strategies between the SIPP and the American Community Survey (ACS), which serves as the data source for the CMS estimates. The SIPP intentionally oversamples low-income adults to assess their participation in federal social service programs while the ACS samples a broader swath of the population. Further, the CMS estimates are from more recent time periods (2012-2016) while the SIPP is from older years (2001, 2004, 2008). The recent growth of the Asian undocumented population may have contributed to the lower proportion in our sample. Finally, the ACS does not directly ask about documented status and researchers use imputation techniques to estimate the likely undocumented population. The imputation technique introduces uncertainty into ACS estimates, particular for smaller samples like Asian immigrants.

We also found the demographic patterns of undocumented immigrants interesting, but we are unable to validate them with other studies that use the SIPP or other data sources. We are not aware of any other studies that have used the SIPP to examine undocumented Asians in detail. Further, we are not aware of other studies with detailed migration information that has examined the Asian undocumented population (e.g, California Health Interview Survey, Los Angeles Family and Neighborhood Survey).

**3. The rates of health insurance coverage seem low to me, even for the period. 20% of USB Asians without insurance and 38% of USB Hispanics? Does your sample include children or is it limited to adults?**

Our sample is limited to adults 18 and older. We thank the reviewer for pointing this out. We caught a coding error, which raised the rates of health insurance coverage to 72% for USB Latinos and 84% for USB Asians. Fortunately, this coding error didn’t drastically change the patterns of the results. This brings our estimates more in line with other estimates. For example, the 2005 Current Population Survey (CPS) show that in 2004, 32.7% of Latinos and 16.8% of Asians in the United States lacked health insurance (DeNavas-Walt, Proctor, and Lee 2005).

**4. Self-rated health is well known to be biased by Spanish language. Is it possible to control for language in which the survey was answered, esp among Latinos? If not, can you speculate about how this bias may relate to legal status and the findings for SRH?**

The review is correct that the self-rated health measure may be biased by language use among Latinos. This may have underestimated differences between undocumented immigrants and the US-born if more undocumented immigrants took the survey in Spanish and choose the fair/poor responses for self-rated health (Viruell-Fuentes et al., 2011). It is not clear that the same is true for Asians, however. Asian immigrants are no less likely than US-Born Asians to choose extreme values for self-rated health (Erosheva et al., 2007).

The SIPP was not administered in Spanish, but the 2004 and 2008 waves have a question about home language use. We did not include this variable in our analysis because it was not asked in 2001. The similar patterns between the self-rated health and disability outcomes, however, suggest to us that language biases are not a major concern for our self-rated health outcome.

**5. Even though I am familiar with other studies of this topic, I still find myself surprised by the result for health status of Latinos. Clearly there is selectivity at work: either into the sample or into the sampling universe (through in/out migration). What are the authors' thoughts on whether SIPP undoc immigrants represent all undoc immigrants? Who is not sampled, and who is not participating in the SIPP?**

As previously discussed, the SIPP oversamples low-income populations. As a result, the demographic trends in the SIPP Latino undocumented population do not completely align with those from the American Community Survey (Capps et al., 2013), the only other national source of data that has been used to produce estimates of the demographic characteristics of undocumented immigrants. While the ACS estimates are for the entire unauthorized population, immigrants from Latin America comprise the large majority of the undocumented and contribute an outsized influence on the overall trends in that dataset. The ACS and SIPP undocumented samples have comparable levels of employment and poverty level. The two samples diverge in marital status, gender breakdown, and US duration. In the ACS, 60% of undocumented immigrants are married versus 46% in the SIPP. The SIPP has fewer undocumented women (47% of the ACS are female versus 40%).

Despite the unique sample, the physical health patterns we found in the SIPP align with other studies. In the 2009 California Health Interview Survey (CHIS), undocumented immigrants had comparable or lower prevalence of diabetes, high blood pressure, asthma, and smoking compared to US-born and other immigrant groups. The only health measure in which they displayed a higher prevalence was obesity (Wallace et al., 2013). Hamilton et al., 2019) conducted a review of 12 studies and did not find undocumented immigrants to have poorer health than documented immigrants in the vast majority of 45 different measures, including self-reports of diagnoses and symptoms, obesity, blood pressure, inflammation, and birth outcomes. In over 20% of these measures, undocumented immigrants had superior outcomes.

We have made a note about the SIPP sampling in the Discussion, under our Limitations.

**6. Check line 124: non-citizens...were further asked whether they have adjusted to LPR status. Do you mean refugees?**

This was not a typo in the text. This was the sequence of questions in the SIPP. Non-citizens were asked if they adjusted to LPR status.

**Reviewer #3:**

**1. In the abstract (line 15), it is better to use the full name of SIPP for the people who are not familiar with this dataset.**

We have made this change.

**2. Is there any relationship among three dependent variables (self-rated health, disability, and current health insurance coverage)? If so, it might be helpful to explain the differences in patterns between Latino and Asian groups**

While several studies show that lack of health insurance is associated with poorer physical health outcomes (Franks et al., 1993; Kasper et al., 2000; McWilliams et al., 2004), among the general population, this relationship does not appear to be as robust for either Asians or Latinos. Using the National Latino and Asian American Survey, Alegria et al found no difference in either self-rated health or disability between those who are insured and those who have private insurance (Alegría et al., 2006). We therefore base our hypotheses based on what we expect given the differences in privileges, resources, and incorporation among the different immigration status, as opposed what one outcome (ie, health insurance) might reveal about another (ie, self-rated health).

**3. In line 91, you mention the order from low (undocumented) to high (US-born) which may make readers feel uncomfortable. They are different immigration statuses, but they have no ranks.**

Our intent was not to rank immigration statuses, but to order them according to a hierarchy of rights, privileges, and incorporation. This approach is guided by the theoretical literature on immigrant incorporation (Bean, 2011), which argues against binary distinction between documented and undocumented immigrants. Instead, immigrant status follows a gradient in which US-born citizens are the most privileged group, while other are the legally vulnerable and socially excluded. We try to make this clearer in the Introduction (lines 64-78).

**4. Can you explain more why you do cross-sectional analysis instead of longitudinal analysis since you have a longitudinal dataset? Is it related to the small sample of the undocumented Asians?**

While the SIPP is a longitudinal dataset (each panel is interviewed repeatedly over a 2.5-4 year period), our analysis is cross-sectional because migration and health information are only asked at one point during the survey data collection period (modules 3 and 6, respectively). We have added this clarification point in the methods.

**5. Can you talk more about your imputation method (line 131-134) maybe in the footnote?**

We have added more information about our imputation technique in the manuscript as a response to this comment and Reviewer 1’s Comment #6.

We used Stata’s mi command, which uses multiple chained equations (MICE) to estimate respondent-level missing data on all variables. This approach assumes that variables are missing at random (MAR) and that other variables in the dataset can be used to predict the missing variable. We used MICE to first handle all missing data besides migration information. We averaged across ten imputed datasets to create a single dataset that was only missing migration information. We conducted MICE again for the migration information only and created ten datasets with imputed migration information that were combined in our analyses using Rubin’s rules.

**6. Can you show the differences in descriptive analysis between non-missing data and missing data?**

The table below compares the samples with complete and missing data for migration information. Approximately 16% of the sample was missing on migration information. There are differences between the samples on several demographic characteristics, such as age, education, gender, marital status, education, number in household, income to poverty. These patterns suggest to us that the data is MAR and that MICE is an appropriate approach to handling missing data.

|  | Latino | | Asian | |
| --- | --- | --- | --- | --- |
|  | Missing | Non-missing | Missing | Non-missing |
| Mean Age | 37.7 | 39.2 | 42.5 | 46.7 |
| Male | 45.6 | 49.6 | 50 | 52.5 |
| Married | 47.7 | 51.8 | 43.4 | 55.7 |
| Education |  |  |  |  |
| Less than HS | 38.9 | 34.9 | 10.8 | 10.1 |
| HS grad | 32.2 | 29.1 | 32.3 | 28.7 |
| Some College | 21.4 | 26.2 | 33.1 | 34.2 |
| Bachelor's and More | 7.5 | 9.8 | 23.8 | 27 |
| Region |  |  |  |  |
| Northeast/Midwest | 22.6 | 22.6 | 40.9 | 44.1 |
| South | 34.9 | 35.8 | 36.9 | 36.1 |
| West | 42.5 | 41.6 | 22.2 | 19.8 |
| Number in hh | 4.5 | 3.8 | 3.2 | 2.8 |
| Number of minor kids | 1 | 0.95 | 0.52 | 0.56 |
| Working | 68 | 69.8 | 66.7 | 68.4 |
| Income to Poverty | 2.58 | 2.82 | 4.1 | 4.3 |

**7. There are some interesting findings in your results (such as line 167-168, line 195-196), can you provide more discussion about these results in your discussion part instead of repeating the results?**

We have added more interpretation to our Discussion, especially about positive health selection and how this may operate differently for Asians and Latinos.

**8. What are the implications of your study with regards to the immigration or health policy or other community programs?**

Undocumented immigrants may not have worse self-rated health or disability, but their insurance coverage – especially for Latino undocumented immigrants - is still very low. This likely reflects their ineligibility for Medicaid and other federal programs. While their positive health profiles may indicate lower need and utilization of medical care, their low insurance coverage poses a health risk in the long-term. As we note now in the text, as immigrants live longer in the United States, the prevalence of poor self-rated health and disability increases, suggesting that undocumented immigrants may not have access to care when they need it. Several municipalities have implemented city or county insurance plans for undocumented immigrants ineligible for Medicaid, providing a crucial resource for medical access.

Alegría, M., Cao, Z., McGuire, T. G., Ojeda, V. D., Sribney, B., Woo, M., & Takeuchi, D. (2006). Health insurance coverage for vulnerable populations: Contrasting Asian Americans and Latinos in the United States. *Inquiry*. https://doi.org/10.5034/inquiryjrnl_43.3.231

Capps, R., Bachmeier, J. D., Fix, M., & Hook, J. Van. (2013). A demographic, socioeconomic, and health coverage profile of unauthorized immigrants in the United States. *Migration Policy Institute Issue Brief*.

Erosheva, E., Walton, E. C., & Takeuchi, D. T. (2007). Self-rated health among foreign- and U.S.-born Asian Americans: a test of comparability. *Medical Care*, *45*(1), 80–87. https://doi.org/10.1097/01.mlr.0000241114.90614.9c

Franks, P., Clancy, C. M., Gold, M. R., & Nutting, P. A. (1993). Health insurance and subjective health status: Data from the 1987 National Medical Expenditure Survey. *American Journal of Public Health*. https://doi.org/10.2105/AJPH.83.9.1295

Hamilton, E. R., Hale, J. M., & Savinar, R. (2019). Immigrant Legal Status and Health: Legal Status Disparities in Chronic Conditions and Musculoskeletal Pain Among Mexican-Born Farm Workers in the United States. In *Demography*. https://doi.org/10.1007/s13524-018-0746-8

Kasper, J. D., Giovannini, T. A., & Hoffman, C. (2000). Gaining and losing health insurance: Strengthening the evidence for effects on access to care and health outcomes. *Medical Care Research and Review*. https://doi.org/10.1177/107755870005700302

McWilliams, J. M., Zaslavsky, A. M., Meara, E., & Ayanian, J. Z. (2004). Health insurance coverage and mortality among the near-elderly. *Health Affairs*. https://doi.org/10.1377/hlthaff.23.4.223

Viruell-Fuentes, E. A., Morenoff, J. D., Williams, D. R., & House, J. S. (2011). Language of Interview, Self-Rated Health, and the Other Latino Health Puzzle. *American Journal of Public Health*, *101*(7), 1306–1313. https://doi.org/10.2105/AJPH.2009.175455

Wallace, S. P., Torres, J. M., Nobari, T., & Pourat, N. (2013). Undocumented and Uninsured: Barriers to Affordable Care for Immigrant Populations. *The Commonwealth Fund*.
