# Supplementary material for: Comparing immigration status and health patterns between Latinos and Asians: Evidence from the Survey of Income and Program Participation
Source: PLoS One. 2021 Feb 2;16(2):e0246239. doi: 10.1371/journal.pone.0246239 (PMC7853486; doi:10.1371/journal.pone.0246239)
Supplement: Attachment — Submitted filename: Response to Reviews R&R II 12_7_20 submitted.docx [file pone.0246239.s002.docx]

**Reviewer #2**

**One argument the authors make for why their estimates differ from ACS is that the SIPP oversamples low-income households, but the authors are using survey weights to adjust for sampling, so sampling should not affect the estimates, is that correct?**

While we use the weights, they may not produce a representative sample of Asian immigrants. The SIPP is meant to be nationally representative and the weights are not formulated for specific subgroups, such as Asian immigrants.

**Why do the authors expect a legal status gradient in health, with better outcomes for more legally privileged and vice versa, when there is no support for this in the extant literature? Is the argument that the theory is correct and the extant data/analysis is wrong? If so, then the authors should return to this in the discussion and clarify (more directly than they do currently) how their results contribute to this literature. Does positive health selection mean the theory is wrong or is that a data problem? Others have speculated and tried to consider positive health selection as a mechanism for the results -- what are next steps for this area of research in terms of disentangling this paradox? What do the authors say to people who read this and say "oh, so undoc status isn't so bad after all, look all these undoc people have great health!"**

While there have not been many papers that have looked at the full range of immigration statuses as we do, we think there is enough indirect empirical evidence to suggest that a legal status gradient in health could exist. Citizens have better mental health than non-citizens and the US-born (Gee et al., 2016). In California data, naturalized citizens and non-citizens were statistically significantly more likely to report fair/poor health compared to U.S.-born citizens in some Asian ethnicities (Bacong, 2020). There are also gradients in social risk factors related to health outcomes. Non-LPR Latinos have higher food insecurity than naturalized and LPR Latinos (Walsemann et al., 2017). In educational outcomes, undocumented immigrants show a disadvantage in college completion and enrollment compared to citizens (Patler, 2018). Our argument is not that the extant data is wrong, but incomplete. Our data is from a large survey, has detailed migration information, considers multiple immigration statuses, examines Latinos and Asian separately, and looks at several health outcomes.

We have revised our theoretical discussion to interpret our results through the broader lens of the Immigrant Health Paradox instead of focusing solely on health selection. We are not arguing that that the notion of immigration status is Fundamental Cause of Disease is incorrect, but rather that it needs to be incorporated with other well-known empirical patterns and theories of immigration health, such as the Immigrant Health Paradox. There is a similar discussion in the sociological immigration literature of whether immigration status is a “master status” that is more consequential than all others in determining immigrant incorporation (Gleeson & Gonzales, 2012) or whether immigration status is one of several intersectional social identities (Enriquez, 2017).

When we take this perspective, we hope the conclusion is not that undocumented status is not harmful to health, but rather to bolster the sources of resilience that keep immigrants healthy in the face of social risk factors. Our findings also counter the narrative that undocumented immigrants drain public resources. In fact, they are healthier than the average American.

**The authors addressed the concern about LNI visa overstayers by coding any LNI with more than 6years in the US as a visa overstayer, and now their undoc Asian sample is better educated than any other of the Asian groups. This seems unlikely to me: 62% of Asian undoc are college educated? I am wondering if the authors are selecting on grad students, or students who enter as BAs and then go to grad school-- a group that has fewer possibilities for adjustment of status before 6 years (as do refugees or temporary workers, who can be sponsored by their employers). Can the authors do something a little more sophisticated with the SIPP data, something akin to what is done with ACS, to impute who is an LNI and who is an overstayer among Asian LNIs with varying years of residence in the US? For instance, if the person is a grad student, foreign born, and in the US for 6+ years, don't code them as undoc? Can the authors tell in the data what kind of LNI visa a person entered with -- if tourist the timeline is different than if refugee, student, fiance, or worker. It seems that, at a minimum, the paper should present results showing sensitivity to these choices, especially since the authors want this paper to contribute by describing this heretofore unseen population (as written in the response to reviewer 3, who suggested taking out characteristics in Table 1 that do not appear in the regressions). In addition, the authors should explain these choices to the reader, as another contribution is to establish procedures for identifying and studying the Asian undoc population in survey data.**

We use a series of questions to code our migration categories. The first asks about citizenship and we code respondents as citizens or non-citizens. For non-citizens, we use the following two questions to code them into detailed immigration categories. The first asks: When [fill TEMPNAME] moved to the United States to live, what was [fill PTEMPNAME] immigration status? There are six responses: (1) Immediate relative or family sponsored permanent resident (2) Employment-based permanent resident (3) Other permanent resident (4) Granted refugee status or granted asylum (5) Non-immigrant (e.g., diplomatic, student, business, or tourist visa) (6) Other. The second question is: Has [fill PTEMPNAME] status been changed to permanent resident? (1) Yes (2) No.

If a foreign-born non-citizen respondent answers 1, 2, 3, in the first question, they are coded as LPR. If they answer 4 or 5 and answer Yes to the second question, they are coded as LPR. If they answer 4 or 5 and No to the second question, they are coded as LNI if they have been in the United States for 5 years and under and coded as undocumented if they have been to the United States for more than 5 years, with the reasoning being that long term LNIs are visa over stayers. We decided on a five year cut-off point because that is the minimum residency requirement for refugees before they can adjust their status.

The reviewer is concerned that we may inadvertently be categorizing graduate students with over 5 years of US residence as undocumented. Unfortunately, non-immigrants are not categorized by specific type so we do not know whether they are on a student visa, tourist visa, or some other temporary visa. In the five year period before our data time period (1996-2003), student visas comprised between 3-5% of all temporary admissions from Asia (DHS Immigration Yearbook), suggesting that long-term student visa holders are a small minority of LNIs. Nonetheless, we make a note of this in the Discussion section as a limitation of our findings.

We conducted alternate analyses where all non-citizen respondents who were coded as 4 or 5 were coded as LNI, regardless of their duration of US residence. The descriptive tables are below. We could not disclose the descriptive statistics for the LNIs when long-term LNIs are coded as undocumented because of cell size requirements for restricted data. When long-term LNIs are not included, well over half of the undocumented have college degrees (58.4%).

Table 1. Long-term LNIs coded as LNIs and as Undocumented

|  | Asians: No Long Term LNI in Undoc | |  | Asians: Long-term LNI as Undoc |
| --- | --- | --- | --- | --- |
|  | FB Non-Im | FB Undoc. |  | FB Undoc |
| Years of Education |  |  |  |  |
| Less than HS | 9.7% | 14.3% |  | 10.3% |
| High School Grad | 13.1% | 11.4% |  | 12.7% |
| Some College | 15.0% | 16.0% |  | 14.9% |
| College Graduate | 62.2% | 58.4% |  | 62.1% |

The migration information in the SIPP is the most detailed information available in secondary data sources. Researchers who do imputations in the ACS do so because they lack the precise migration information in the SIPP. Further, the ACS does not do imputations that are specific to Asian immigrants and it’s unknown whether the same approaches work equally well for all racial/ethnic undocumented groups. In other work, we find that these techniques work well for Latino immigrant groups, but less so for Asian immigrants.

**Given the quite different age profiles of the groups, and the sensitivity of health conditions, especially disability, to age, it would seem appropriate to consider whether age should be included as a non-linear term in the models.**

We did not include age as a quadratic term in our model. We acknowledge that our results do not fully control for age differences, especially for the FB naturalized group, which was older than the other groups. Despite this, we did not find the FB naturalized group to differ significantly from the referent group when age was included as a linear term.

Going back to the data will to make this in the model may be difficult; our access to the restricted data closed this fall. If the editor really wishes to see these updated models with the quadratic age term, we can re-start the project and make the appropriate updates and check on this prior to publication, but this could delay the review process.

**Reviewer #3**

**All comments have been addressed. That would be great if you could discuss the policy context or insights from 2001 to 2008, which might be different from what we are now in the U.S. How the results from those years may inform current or further studies could be discussed more as well**

We have added some discussion of the policy context in our Discussion. We discuss how this era was before the era of intense internal immigration enforcement (i.e. deportations) that began with the Obama Administration and continued through the Trump Administration. We might expect the health of undocumented immigrants to decline relative to US-born citizens if internal immigration enforcement stressors take a toll on their well-being. Nonetheless, studies from this era can inform current and future studies by providing baseline trends in the pre-enforcement, high undocumented immigrant population growth era.

**References**

Bacong, A. M. (2020). Heterogeneity in the Association of Citizenship Status on Self-Rated Health Among Asians in California. *Journal of Immigrant and Minority Health*. https://doi.org/10.1007/s10903-020-01039-w

Enriquez, L. E. (2017). A ‘master status’ or the ‘final straw’? Assessing the role of immigration status in Latino undocumented youths’ pathways out of school. *Journal of Ethnic and Migration Studies*, *43*(9), 1526–1543. https://doi.org/10.1080/1369183X.2016.1235483

Gee, G. C., Morey, B. N., Walsemann, K. M., Ro, A., & Takeuchi, D. T. (2016). Citizenship as Privilege and Social Identity. *American Behavioral Scientist*. https://doi.org/10.1177/0002764216632834

Gleeson, S., & Gonzales, R. G. (2012). When Do Papers Matter? An Institutional Analysis of Undocumented Life in the United States. *International Migration*, *50*(4), 1–19. https://doi.org/https://doi.org/10.1111/j.1468-2435.2011.00726.x

Patler, C. (2018). Citizen Advantage, Undocumented Disadvantage, or Both? The Comparative Educational Outcomes of Second and 1.5‐Generation Latino Young Adults. *International Migration Review*. https://doi.org/10.1111/imre.12347

Walsemann, K. M., Ro, A., & Gee, G. C. (2017). Trends in food insecurity among California residents from 2001 to 2011: Inequities at the intersection of immigration status and ethnicity. *Preventive Medicine*, *105*, 142–148. https://doi.org/https://doi.org/10.1016/j.ypmed.2017.09.007
